# Idiopathic fibrosing pancreatitis: A rare cause of obstructive jaundice

**DOI:** 10.1002/jpr3.12018

**Published:** 2024-01-30

**Authors:** Mindy Huynh, Rodolfo Silva, Nikhil Thiruvengadam, Kalyan Parashette

**Affiliations:** ^1^ Pediatrics, Pediatric Gastroenterology Loma Linda University Children's Hospital Loma Linda California USA; ^2^ Gastroenterology Loma Linda University Medical Center Loma Linda California USA

**Keywords:** cholestasis, ERCP, idiopathic fibrosing pancreatitis, jaundice

## Abstract

Jaundice is an important physiologic manifestation of both benign and insidious diseases. We report on the case of an 11‐year‐old male who presented with diffuse pruritis, jaundice, and later abdominal pain. Initial work‐up revealed an obstructive cholestatic pattern, warranting investigation for structural anomalies. Extensive imaging revealed a lesion on the pancreatic head, and biopsy of the lesion confirmed the diagnosis of idiopathic fibrosing pancreatitis (IFP). Temporary stenting of the common bile duct successfully treated our patient's symptomatic IFP.

## 1 INTRODUCTION

Pancreatitis is a pathologic state of the pancreas caused by inflammation or injury. It can result in acute recurrent pancreatitis (ARP) or progression to chronic pancreatitis (CP) with functional and structural deficits.^1^ While smoking, alcohol use, and gallstones are major risk factors for pancreatitis in adults, these factors are not as common in children. Genetics, obesity, and obstructive factors are the most common causes of ARP and CP in children.^2^ Other disease processes include idiopathic, autoimmune, and toxin‐driven damage.

## 2 CASE REPORT

The patient is an 11‐year‐old male with obesity, eczema, and seasonal allergies who presented with 3 days of pruritus and 1 day of rapidly worsening jaundice and scleral icterus. Three weeks before his presentation, he had an upper respiratory infection. His mother had symptomatic cholelithiasis during pregnancy, and she was now status post cholecystectomy. His father noted a significant family history of adult‐onset pancreatitis and pancreatic cancer without a precise genetic diagnosis—this includes a paternal grandmother, maternal grandfather, and paternal aunt.

His initial workup revealed direct hyperbilirubinemia (direct bilirubin: 18.4 mg/dL, total bilirubin: 22.6 mg/dL), AST of 51 U/L, ALT of 40 U/L, alkaline phosphatase of 730 U/L, and a negative hepatitis A, B, and C screen. Lipase was 69 U/L (normal 73–393 U/L). An abdominal ultrasound showed dilation of the common bile duct (CBD) up to 11.4 mm, mild intrahepatic duct dilation, and distention of the gallbladder. The pancreas was incompletely visualized, though no other masses were noted. These findings were concerning for biliary obstruction. He subsequently developed abdominal tenderness over the right upper quadrant. Given the dilation of the CBD and intrahepatic duct, there was concern for a small distal stone. Acute pancreatitis was less likely given a normal lipase. At this time, endoscopic evaluation was pursued first to evaluate for choledocholithiasis.

**Figure 1:**
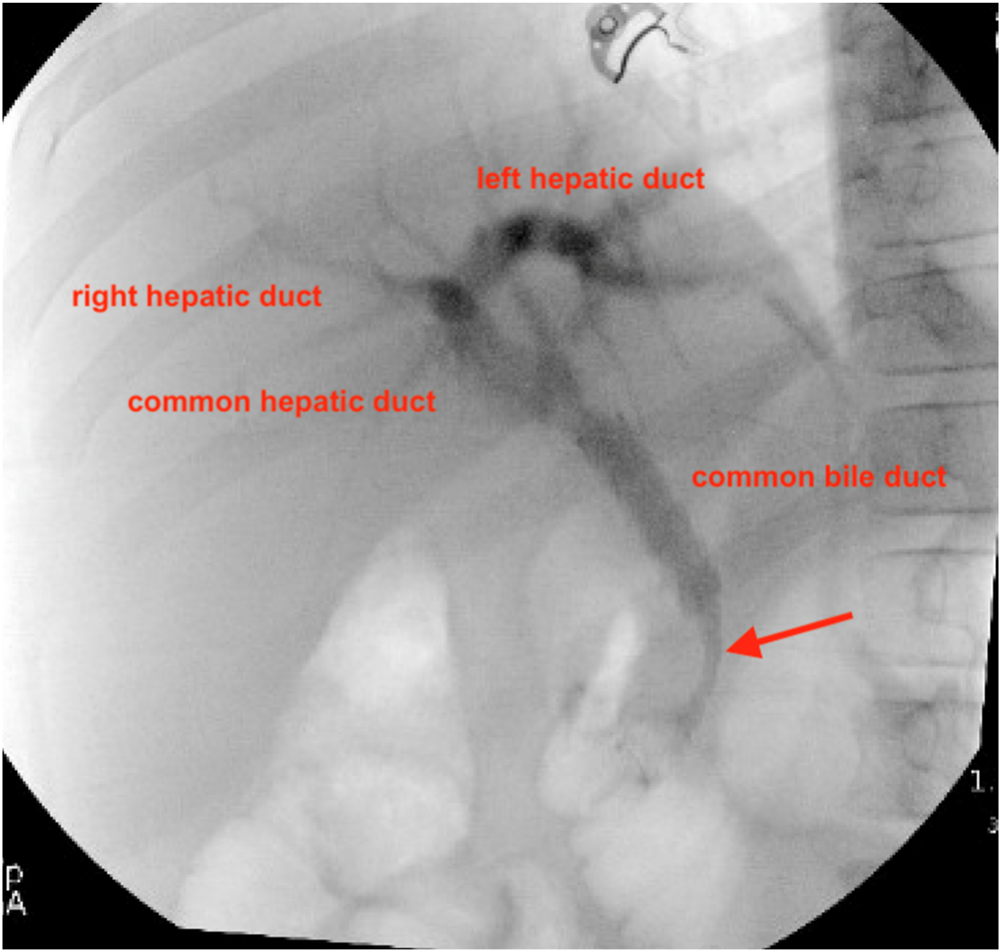
Endoscopic retrograde cholangiopancreatography (ERCP). ERCP shows distal common bile duct narrowing (arrow) and proximal dilation.

Endoscopic ultrasound (EUS) revealed a high‐grade intrapancreatic bile duct stricture that initially raised concern for CP or pancreatic adenocarcinoma. The stricture was slightly hypoechoic, with other surrounding hyperechoic foci—where fine‐needle biopsy (FNB) was obtained with a 22‐gauge needle, which showed rare epithelial cells with prominent nucleoli, mixed inflammatory cells, and fibrosis—consistent with reactive versus reparative change. He underwent endoscopic retrograde cholangiopancreatography (ERCP), which showed normal‐appearing intrahepatic biliary ducts and a dilated CBD (Figure 1). A 10 mm by 8 cm fully covered self‐expanding metal stent (FCSEMS) was placed within the CBD. Distal CBD brushing specimens showed no identifiable dysplasia or malignancy.

**Figure 2:**
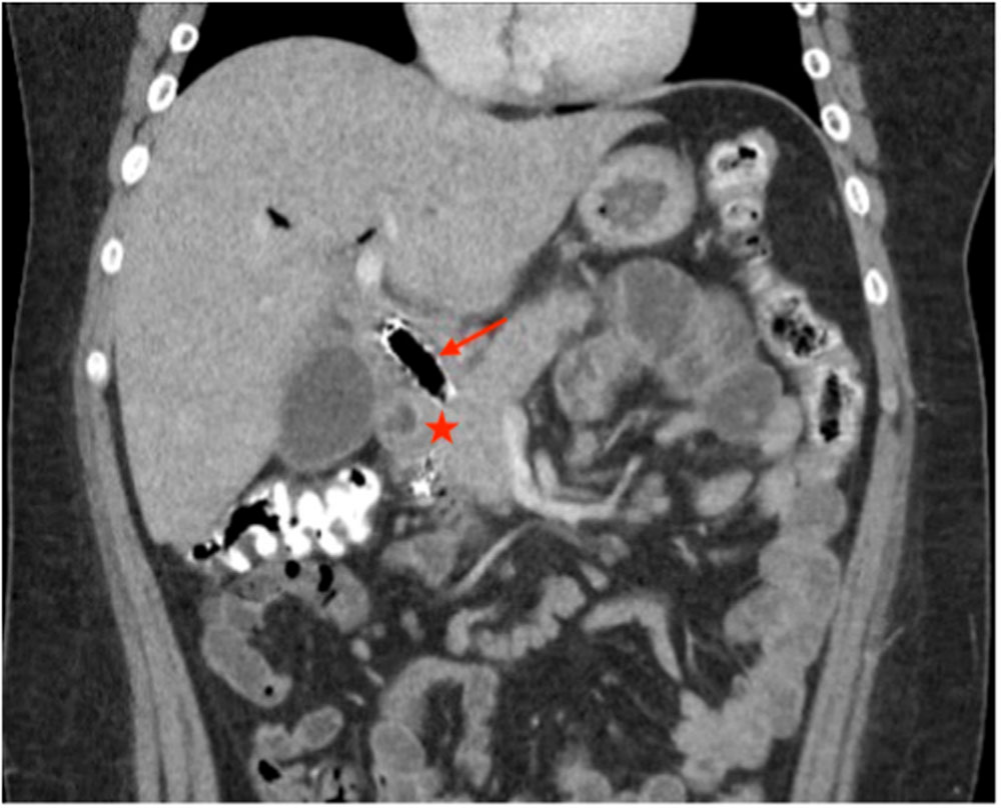
Computed tomography abdomen with contrast. Hypo‐enhanced lesion in head of pancreas (star) with mass effect on the bile duct stent (arrow). Pancreatic head mass measures up to 14 mm.

Following his ERCP and EUS, a computed tomography (CT) abdomen study showed a hypo‐enhancing lesion of the pancreatic head with a mass effect on the adjacent stent (Figure 2). The identified lesion increased suspicion of adenocarcinoma. Pancreatic tumor markers were sent and returned within normal ranges, including serum carcinoembryonic antigen (CEA) of 1.9 ng/mL and cancer antigen 125 (CA‐125) of 15.6 Units/mL. Chromogranin A was normal at 33 ng/mL and argued against carcinoid or neuroendocrine tumors. Autoimmune work up included antinuclear antibody, smooth muscle antibody, anti‐liver kidney microsomal antibody, total immunoglobulin G, and immunoglobulin G subtype 4 (IgG4)—these were also unremarkable, making autoimmune pancreatitis (AIP) type 1 less likely.

**Figure 3:**
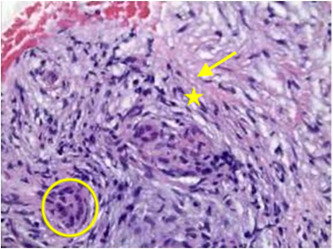
Fine‐needle biopsy of pancreatic head. Stromal fibrosis (arrow) surrounding entrapped ducts (star) and some acinar tissue (circle), with associated chronic inflammatory cells, rare neutrophils, and a few eosinophils. There are few bland spindled and collagenized tissue, with admixed rare pancreatic acini and ductal elements. S100 stain was negative.

He underwent repeat ERCP and EUS 6 days later. The ERCP showed residual distal CBD stenosis despite the FCSEMS, with proximal CBD dilation to 8 mm. The EUS redemonstrated an abnormal pancreas with diffuse hypoechoic foci, stranding, and lobularity. There was a slightly hypoechoic 1.8 cm × 1.3 cm area adjacent to the stricture. Repeat FNB of this site showed stromal fibrosis and chronic inflammatory cells (Figure 3). Staining for S100, which can be associated with adenocarcinoma, was negative. There were no infectious organisms seen, and significant acute inflammation was lacking. IgG4 and plasma cell stains were not performed, however, this may have been useful to rule out AIP further. Given the bland appearance of the tissue, normal pancreatic lipase, and low stool pancreatic elastase <40 U/L, a reactive or idiopathic obstructive process such as idiopathic fibrosing CP was favored. The Hereditary Pancreatitis Panel testing for mutations of PRSS1, CFTR, CTRC, and SPINK1 was negative.

During his hospital stay, his blood cell counts and liver function tests were trended. The direct bilirubin level trended downward to 6.0 mg/dL over 8 days. His ALT and AST began to climb towards the end of the hospitalization to 526 and 421 U/L, respectively, likely due to his multiple ERCP procedures. One month following discharge, he had an MRI abdomen, which demonstrated similar positioning of the CBD stent, without biliary duct dilation and a normal appearing pancreas. Five months after discharge, he underwent ERCP and EUS for stent removal and biliary sludge evacuation. The EUS showed mild lobularity and atrophy of the pancreatic head with improved stranding. The bile duct was dilated at 7.7 mm, and the distal CBD stricture had resolved.

Following his hospitalization, the patient made significant lifestyle changes to help lose weight. While his initial fecal elastase was low, his initial fat‐soluble vitamin levels were normal. He denied diarrhea, steatorrhea, or other exocrine dysfunction at follow‐up visits, therefore pancreatic enzyme supplementation was deferred in the interim. Approximately 1 year since his stent removal, he has not had a recurrence of jaundice or developed new symptoms of pancreatic insufficiency. His liver enzymes have remained normal.

## 3 DISCUSSION

Idiopathic fibrosing pancreatitis (IFP) is a rare obstructive disease characterized by extrinsic compression of the distal CBD, often with a mass at the pancreatic head.^3^, ^4^ Patients usually present with jaundice and abdominal pain, developing over days to weeks. Later in the disease course, patients may develop exocrine pancreatic insufficiency, however, endocrine function is usually conserved.^5^

IFP and autoimmune pancreatitis (AIP) are difficult to distinguish clinically due to their many overlapping symptoms.^5^, ^6^ Making a diagnosis between IFP and AIP, particularly type 2, may be especially challenging. An obstructive jaundice picture is shared, however, abdominal pain is less prominent in AIP, especially with later presentation.^6^ Imaging and biopsy can be used to differentiate and confirm the diagnosis. Focal mass effect and atrophy are characteristic of IFP, while in AIP the pancreas is diffusely enlarged. Focal pancreatic duct narrowing with upstream ductal dilation can be seen in both.^6^ Biopsy of AIP would reveal more lymphocytic infiltration or granulocytic lesions and an elevated serum IgG4; storiform fibrosis and obliterative phlebitis are also diagnostic hallmarks.^4^ Notably, type II AIP can appear to be more consistent with IFP than type I AIP, including lack of positive IgG4 and pancreatic enlargement or other focal findings on parenchymal imaging.^6^ AIP is responsive to glucocorticoid therapy.

Traditionally, IFP has been treated with surgical decompression by bilioenteric bypass or sphincteroplasty. In 1998, Sylvester et al. published one of the first case series in which endoscopic drainage was successful in three of four patients with IFP.^5^ Temporary stenting can allow drainage while the mass effect of the pancreas is allowed to self‐resolve.^7^ There is ongoing research into other conservative measures, including steroids and ursodeoxycholic acid, before invasive management.^8^ While the incidence of IFP is extremely low, with about 47 cases described in the literature, many of these patients eventually developed long‐term pancreatic dysfunction or atrophy at or soon after diagnosis, requiring enzyme replacement.^4^, ^7^

Though a rare diagnosis, IFP should be considered in the differential diagnosis in pediatric obstructive jaundice, even in the absence of abdominal pain as was initially seen in our patient. Nonsurgical management should be pursued in otherwise stable patients without evidence of fulminant liver damage. Close follow‐up with weight assessment every 6–12 months is crucial to monitor pancreatic function and recovery. Stool elastase and serum fat‐soluble vitamin levels should be followed annually if clinically indicated.

## CONFLICT OF INTEREST STATEMENT

The authors declare no conflict of interest.

## ETHICS STATEMENT

Informed patient consent was obtained from parent/guardian for publication of the case.
